# Cardiac arrest in a 36 year-old after trigger point injection with lidocaine: Case report

**DOI:** 10.1016/j.inpm.2022.100077

**Published:** 2022-03-02

**Authors:** Jacob Hattenbach, Haruki Ishii, Samantha Mastanduno, Tracy Espiritu McKay

**Affiliations:** a Department of Rehabilitation Medicine at NYU Grossman School of Medicine, New York, NY, USA; b Department of Physical Medicine and Rehabilitation at Rothman Orthopaedic Institute, New York, NY, USA; c Department of Rehabilitation Medicine at NYU Grossman School of Medicine, NYU Langone Health, New York, NY, USA

**Keywords:** Trigger point injection, Myofascial pain, Lidocaine, Vasovagal syncope, Cardiac arrest

## Abstract

A typical vasovagal response is characterized by bradycardia and paradoxical vasodilation. It is oftentimes self-limited and spontaneously reversible; however, severe cases can result in significant complications. This report describes a case of profound vasovagal syncope with subsequent cardiac arrest in the setting of receiving a trigger point injection. This patient presented to an outpatient clinic for ultrasound-guided left trapezius, levator scapulae, and rhomboid trigger point injections for relief of myofascial pain. One milliliter of 1% lidocaine was injected into the first trigger point when she stated she felt dizzy before becoming unresponsive without palpable peripheral pulses. The patient regained consciousness following cardiopulmonary resuscitation (CPR). Considering the frequency of office based pain procedures, it is important to recognize the potential serious complications associated with procedures frequently thought to be benign.

## 1 Introduction

With the increasing number of interventional and office-based procedures being performed for the treatment of musculoskeletal pain, it is important to consider the potential adverse effects of these procedures. Trigger point injections (TPI) are generally considered safe, and commonly used for treatment of myofascial pain syndrome by inserting a needle into a myofascial trigger point [1]. Oftentimes these injections utilize local anesthetics which are thought to alleviate nerve irritation and referred pain by inactivating tight muscular bands [2]. However, rare TPI complications have been reported, including pneumothorax, intrathecal injection, epidural abscess, and skeletal muscle toxicity [3]. Occasionally injections may also cause syncope [4]. Normally, vasovagal response is self-limited and spontaneously reversible; however, severe vasovagal responses can result in cardiac arrest [5]. In this case, we describe profound vasovagal syncope with subsequent cardiac arrest in the setting of receiving a trigger point injection.

## 2 Case presentation

A 36-year-old female (BMI 29.6, Weight 173 lbs) presented to an outpatient clinic for ultrasound-guided left trapezius, levator scapulae, and rhomboid trigger point injections with 1% lidocaine for relief of myofascial pain. The patient expressed her anxiety about the injection but wanted to proceed if it would mitigate her myofascial pain. Her medical history included hyperlipidemia, anxiety, and depression. She reported taking meloxicam, and denied known allergies or adverse reactions to prior injections. Vital signs were stable, and she was alert, oriented, and able to provide informed consent for the procedure.

Prior to the procedure, the skin was prepped with chlorhexidine, non-latex gloves were utilized, and point of care ultrasound was used to examine upper trapezius, levator scapulae, and rhomboid trigger points. Upon introduction of a 27-gauge 1.5-inch hypodermic needle, ultrasound confirmed proper needle placement and aspiration without blood return, ensuring the needle was intramuscular and not intravascular. One milliliter of preservative free 1% lidocaine without epinephrine was injected into the first trigger point. The needle was removed and inserted into the adjacent trigger point using sterile technique with ultrasound guidance. At this point, the patient stated that she felt dizzy and moved from sitting to lying on her right side. The patient became unresponsive and pale in her face. Jaw thrust was attempted while support staff obtained the automated external defibrillator (AED). No peripheral pulse was palpable and CPR was initiated while emergency services were called. The AED reported an unshockable rhythm. Chest compressions were performed for approximately 1 ​min at which point the patient regained consciousness and pulses returned. She was noted to be diaphoretic and incontinent of urine. After regaining consciousness vital signs were obtained (BP 100/84, Pulse 62, RR 11, SpO2 98%) and electrocardiogram (ECG) was performed which revealed normal sinus rhythm. Upon arrival of EMS, the patient was alert and oriented.

The patient was brought to the emergency department for further workup. In the ED, workup including EKG and cardiac markers were normal. The patient underwent an outpatient cardiac workup and was told this event was thought to be a “severe vasovagal episode.” It is suspected that the patient presented here suffered an adverse reaction to the trigger point injection resulting in a vasovagal episode complicated by seizure activity and cardiac arrest.

## 3 Discussion

Our differential for the adverse events in this case includes vasovagal syncope in response to TPI, local anesthetic systemic toxicity (LAST), and allergic reaction.

### 3.1 Vasovagal syncope

Vasovagal response is characterized by bradycardia and paradoxical vasodilation. This response may include short-term dizziness, nausea and weakness, subsequent loss of consciousness and myoclonic movements**.** Vasovagal response can be caused by a variety of reasons including severe pain, anxiety, fear, and emotional stress, leading to cardioinhibitory response secondary to a sudden activation of parasympathetic activity also known as vagotonia and/or inhibition of sympathetic activity [5]. Afferent fibers to the brainstem can be activated by mechanical and chemical stimuli due to the collapsed left ventricular wall secondary to sudden decreased venous return. These afferent fibers can then increase parasympathetic activity and decrease sympathetic activity, leading to bradycardia and vasodepressor response [5]. Vasovagal response, though its occurrence is not significantly prevalent, can be observed in outpatient procedures such as ultrasound-guided injections. Al-Assam et al. found an overall rate of vasovagal response to be 2.3% for all ultrasound guided musculoskeletal steroid and local anesthetic injections, more commonly in females and less likely in ages greater than 65 years old [6]. As previously mentioned, vasovagal response is typically self-limited and spontaneously reversible; however, severe cases can occasionally result in significant complications, including cardiovascular collapse, shock or cardiac arrest [5]. To our knowledge, there has only been one reported case of lidocaine injections for trigger points leading to cardiac arrest [7].

### 3.2 Local anesthetic systemic toxicity (LAST)

LAST is a very rare complication associated with the use of local anesthetic agents [8] with an estimated incidence of 0.03–0.09%, or 0.27–0.92 episodes per 1000 peripheral nerve blocks [9,10]. The rate that the medication reaches the intravascular compartment depends upon route of administration, most rapidly when administered into the intercostal space, followed by the caudal, epidural, brachial plexus, femoral, and subcutaneous spaces [11]. Injections to highly vascularized sites pose a greater risk of LAST given increased risk of systemic absorption and distribution. Local anesthetics such as lidocaine attach to voltage-gated sodium channels thus inhibiting neuronal ion transfer and depolarization and preventing neuronal transmission [9,11]. Signs and symptoms of CNS toxicity induced by local anesthetic can resemble vasovagal responses. Mild toxicity can occur with plasma levels greater than 5mcg/mL and result in slurred speech, tinnitus, circumoral paresthesia, and feeling faint [11]. Seizures or loss of consciousness can occur at plasma levels above 10mcg/mL. Cardiac arrhythmias, respiratory arrest and cardiac arrest can occur at levels above 20 mcg/mL. Studies have found that compared to other local anesthetics, lidocaine may be less likely to progress rapidly through neurological signs and symptoms to full cardiovascular collapse in cases of toxic dosing [11]. Avoidance of intravascular injection into major vessels and care not to exceed the maximum total dose of anesthetic helps to avoid complications in most patients. Diagnostic ultrasound has been increasingly used to improve the accuracy of needle placement, thus leading to a better safety and efficacy of office-based procedures. Diep et al. conducted a systematic review, assessing the benefit and safety of ultrasound guided interventional procedures for treatment of myofascial pain syndrome, which demonstrated that ultrasound guided procedures resulted in minimal self-limited adverse events, including minor pain and diaphoresis [12]. Additionally, one study found that the use of ultrasound guidance during peripheral nerve blocks led to a reduced incidence of LAST compared to blind injections [8]. Given proper intramuscular needle placement via ultrasound without evidence of intravascular placement with extremely low dose of lidocaine, LAST is exceedingly unlikely the etiology of cardiac arrest in this case.

### 3.3 Allergic reaction

In clinical practice, allergic reaction is not an infrequent event and normally subsides without specific intervention or with use of antihistamine alone or in combination with steroids [13]. Anaphylaxis, a type 1 hypersensitivity response, however, is the most severe type of allergic reaction, which can result in death without immediate care [13]. Anaphylactic reactions secondary to local anesthetic agents are extremely rare and previously reported to be less than 1% [[14], [15], [16], [17]]. Though few cases of allergic reaction were previously reported, allergic reaction is less likely the cause of cardiac arrest seen in the present case given the rarity of lidocaine anaphylaxis and the patient's lack of known allergy to lidocaine.

Although commonly self-limited as stated above, vasovagal response due to emotional stress and anxiety may have played a significant role in the development of cardiac arrest in the present case. In our patient, bradycardia and vasodilation mediated by increased vagal activity, particularly to the heart, and reduced sympathetic activity led to profound hypotension, likely having resulted in cardiac arrest. Vasovagal syncope is one of the subcategories of reflex syncope, the most frequent cause of syncope in any setting and age group [18], and thus it is difficult to foresee, making it challenging to prevent. The hemodynamic collapse during a profound vasovagal response occurs instantly [5]; in the current case, asystole was noted in a matter of seconds after the patient changed position from sitting to lying on her right side. Fortunately, immediate assessment and treatment were given in this case, leading to rapid recovery without obvious complications. Other treatment that resulted in similar rapid recovery with sufficient outcomes in cardiac arrest due to vasovagal reaction in previous case reports includes early recognition, chest compression, manual ventilation with 100% oxygen, rapid hydration, and administration of atropine, ephedrine, phenylephrine, and epinephrine [5,[19], [20], [21], [22]]. A careful history-taking regarding prior fainting or syncope could have decreased the risk of unprepared vasovagal response. With patients who are overly concerned or fearful about TPI, alternative treatment may be more appropriate. Consideration may also be taken to monitor heart rate and pulse oximetry during minor procedures alerting clinicians to any impending adverse events. Attention should also be paid to patient positioning during procedures. Using an examination table instead of a chair may be more prudent allowing clinicians to safely change patient position in case of vasovagal reaction. Topical anesthetics including lidocaine cream may reduce pain during TPI which in turn may decrease the risk of vasovagal response by reducing the emotional stress patients may perceive during the procedure. Lastly, prophylactic anxiolytic agents or IV sedatives should be considered in severe cases of anxiety or history of vasovagal reactions to reduce the risk of adverse events.

## 4 Conclusion

We describe a patient with severe vasovagal cardiac arrest during TPI for treatment of myofascial pain syndrome. The present case is an excellent example, illustrating the importance of recognizing the potential serious complications associated with procedures frequently thought to be benign. A careful history taking and pre-procedural evaluation, early identification of vasovagal response, and prompt response to vasovagal reaction are important measures learned from this case and should be applied by all practitioners.

## Author disclosures

None of the authors involved in the creation of this case report have identified any competing interests. This project was not funded by any organization, and there is no financial incentive for any of the authors.

## Declaration of competing interest

The authors declare that they have no known competing financial interests or personal relationships that could have appeared to influence the work reported in this paper.
